# Stimulation-induced ectopicity and propagation windows in model damaged axons

**DOI:** 10.1186/1471-2202-15-S1-P134

**Published:** 2014-07-21

**Authors:** Mathieu Lachance, André Longtin, Catherine E Morris, Na Yu, Béla Joós

**Affiliations:** 1 Département de physique, Cégep de l’Outaouais – campus Félix-Leclerc, Gatineau, Québec, Canada J8T 7T7; 2 Ottawa-Carleton Physics Institute, University of Ottawa, Ottawa, Ontario, Canada K1N 6N5; 3 Neurosciences, Ottawa Hospital Research Institute, Ottawa, Ontario, Canada K1H 8M5

## Background

Neural tissue injuries render voltage-gated Na^+^ channels (Nav) leaky, thereby altering excitability, disrupting propagation and causing neuropathic pain related ectopic activity [1]. In both recombinant systems and native excitable membranes, membrane damage causes the kinetically-coupled activation and inactivation processes of Nav channels to undergo hyperpolarizing shifts [2]. This damage-intensity dependent change, now called [3] coupled left-shift (CLS), yields a persistent or “subthreshold” Nav window conductance. Previous simulation work involving various degrees of mild CLS has focused on individual nodes of Ranvier or simple propagation models [3-5], leaving open an important question: does mild-injury (small CLS values, pumps functioning well) render propagation-competent but still quiescent axons vulnerable to further impairments as the system attempts to cope with its normal excitatory inputs? We probed this incipient diffuse axonal injury scenario using a 10-node myelinated axon model with dynamic ion gradients and Nernst potentials.

## Results

Fully restabilized axons with mild damage, while they remain quiescent in the absence of stimulation, can abruptly switch behavior after receiving normal impulse traffic from upstream neurons. Because incoming action potentials stress Na^+^ and K^+^ gradients, thereby altering spike thresholds, the damaged nodes of Ranvier can become ectopic signal generators (“ectopic nodes”), emitting neuropathic pain-like (or epileptiform) barrages of impulses. Comparable changes could contribute to diverse acquired sodium channelopathies [6] and to the neuropathic amplification of normally benign sensory inputs [7].

Also, and perhaps counter-intuitively, more intensely damaged sites firing ectopically can be dominated by incoming high-frequency impulse traffic, enabling a “propagation window” of finite lifetime. This intermittent “propagation window” is a robust phenomenon, occurring despite noise, large jitter and the presence of several consecutive ectopic nodes. Complex input spike patterns then propagate with good fidelity as long as their frequencies exceed the ectopic frequency. The faithful propagation of such impulse trains through a damaged zone is likely to complicate diagnosis.
